# Cross-Cultural Adaptation and Psychometric Properties of the Malay Version of the Communication Skills Attitude Scale (CSAS) among Medical Students in Malaysia

**DOI:** 10.3390/ijerph18073778

**Published:** 2021-04-05

**Authors:** Mohamad-Zikri Mohamad-Isa, Mohamed-Syarif Mohamed-Yassin, Siti Fatimah Badlishah-Sham, Noorhida Baharudin, Anis Safura Ramli

**Affiliations:** 1 Department of Primary Care Medicine, Faculty of Medicine, Universiti Teknologi MARA, Jalan Prima Selayang 7, Batu Caves 68100, Malaysia; ag_irkiz@hotmail.com (M.-Z.M.-I.); sfatimah31@uitm.edu.my (S.F.B.-S.); noorhida8229@uitm.edu.my (N.B.); anis014@uitm.edu.my (A.S.R.); 2 Institute of Pathology, Laboratory & Forensic Medicine (I-PPerForM), Universiti Teknologi MARA, Jalan Hospital, Sungai Buloh 47000, Malaysia

**Keywords:** communication skills, cross-cultural adaptation, communication skill attitude scale, Malay version, medical student, medical education, validation study, attitude

## Abstract

Communication is one of the fundamental skills in the medical profession. The Communication Skills Attitude Scale (CSAS) is a widely used questionnaire to measure the attitudes of medical students toward learning communication skills. It has been adapted and translated into many languages. The objective of this study was to adapt and translate the CSAS into the Malay language and determine its psychometric properties in medical students. This is a cross-sectional study involving 218 first-year Universiti Teknologi MARA students. Content validation, cross-cultural adaptation, translation, and face validation of the 26-item CSAS were performed according to established guidelines. Principal component analysis with direct oblimin rotation was used to determine the underlying structure of the CSAS-Malay. The reliability was assessed using Cronbach’s α coefficient for internal consistency and using the intraclass correlation coefficient for the test–retest reliability. Although the contents of the CSAS-Malay and the original version were conceptually equivalent, item 11 was removed during the content validation stage due to a low item content validity index (I-CVI < 1.00). Two subscales were derived from the remaining 25 items, which were the Positive Attitude Scale and the Negative Attitude Scale. Items 1 and 15 were removed due to poor factor loadings. The total variance explained by the final two-factor solution with three items removed was 30.8%. Cronbach’s α coefficients for both the Positive and Negative Attitude Scales in the final questionnaire were 0.815 and 0.614, respectively. It also showed a good reproducibility with intraclass correlation coefficient (ICC) values of 0.725–0.950 for all the items. This study provided preliminary information about the psychometric properties of the CSAS-Malay. The final 23-item questionnaire had a good construct validity, an acceptable internal consistency, and at least a moderate test–retest reproducibility. It can be used to assess the attitudes of medical students toward learning communication skills. Future research to improve the generalizability of the questionnaire should include medical students from other universities with diverse backgrounds.

## 1. Introduction

Communication is one of the fundamental skills in the medical profession. It is defined as the ability to effectively and efficiently convey information to another person [1]. Effective communication between healthcare providers, patients, and their families can improve the outcomes and satisfaction of patients. Healthcare providers with effective communication skills are able to provide the appropriate information, enabling patients to better understand medical information. Consequently, this increases their adherence to treatment [2]. Effective communication has also been shown to contribute to a reduction in the utilization of healthcare resources and malpractice lawsuits [3].

Competency in communication skills can be developed among medical students using formal communication skills training programs [4]. The importance of good communication skills in medical students and doctors has been highlighted by the Australian Health Practitioner Regulation Agency (AHPRA) and the UK General Medical Council (GMC) [5,6]. In Malaysia, the 2015 Guidelines for Accreditation of the Malaysian undergraduate medical education program by the Malaysian Medical Council (MMC) emphasize that graduates should be able to “Communicate clearly, considerately and sensitively with patients, relatives, colleagues, nurses and other health professionals and the general public” upon completion of their medical course [7].

Interestingly, many studies have shown that the exposure to communication skills teaching can affect the students’ positive or negative attitudes toward learning these skills [8,9,10]. Studies assessing the attitudes of medical students toward learning communication skills showed conflicting results [8,11,12,13]. When they were taught communication skills, some students demonstrated an improved attitude, while others did not [8,11,14,15]. Nevertheless, the AHPRA, GMC, and MMC still recommend that communication skills should be emphasized among medical students. 

In Universiti Teknologi MARA (UiTM), communication skills were taught to the year 1 and year 2 medical students during their Early Clinical Exposure (ECE) classes. Both clinical and nonclinical lecturers were involved in teaching these classes. Communications skills were taught using lectures and roleplay sessions involving simulated patients. 

However, it has been previously documented that there is resistance toward social sciences teaching, including communication skills among medical students [16]. Hence, it is important to identify students’ attitudes toward learning communication skills, and the factors associated with their positive or negative attitudes. These findings may guide the improvement of communication skills teaching. A valid and reliable tool is needed for this purpose.

In 2002, Rees, Sheard, and Davies developed the Communication Skills Attitude Scale (CSAS), which is a widely used questionnaire to measure the attitudes of medical students toward learning communication skills [13]. A qualitative study was conducted by these authors to explore the attitudes of medical students toward learning communication skills. Two attitude-related themes were derived: positive attitude and negative attitude [17]. Based on this, the 26-item CSAS, framed within two subscales, the Positive Attitude Scale (PAS) and the Negative Attitude Scale (NAS), was developed [18]. This questionnaire was designed to assess medical students’ positive or negative attitudes toward learning communication skills. 

Internationally, the CSAS has been adapted and translated into many languages [8,9,11,19,20,21,22,23]. Baharudin et al. adapted and validated the CSAS English version in a cohort of University Teknologi MARA (UiTM) students, but the internal consistency of the NAS was low [24]. The majority of these students speak Malay as their first language. The authors’ hypothesis is that the psychometric properties of the scale would improve if it is adapted and translated into the Malay language among students who speak this language as their mother tongue. Therefore, the aim of this study was to adapt, translate, and validate the original CSAS from English into the Malay language. 

## 2. Materials and Methods

### 2.1. Study Design and Participants

**Figure 1:**
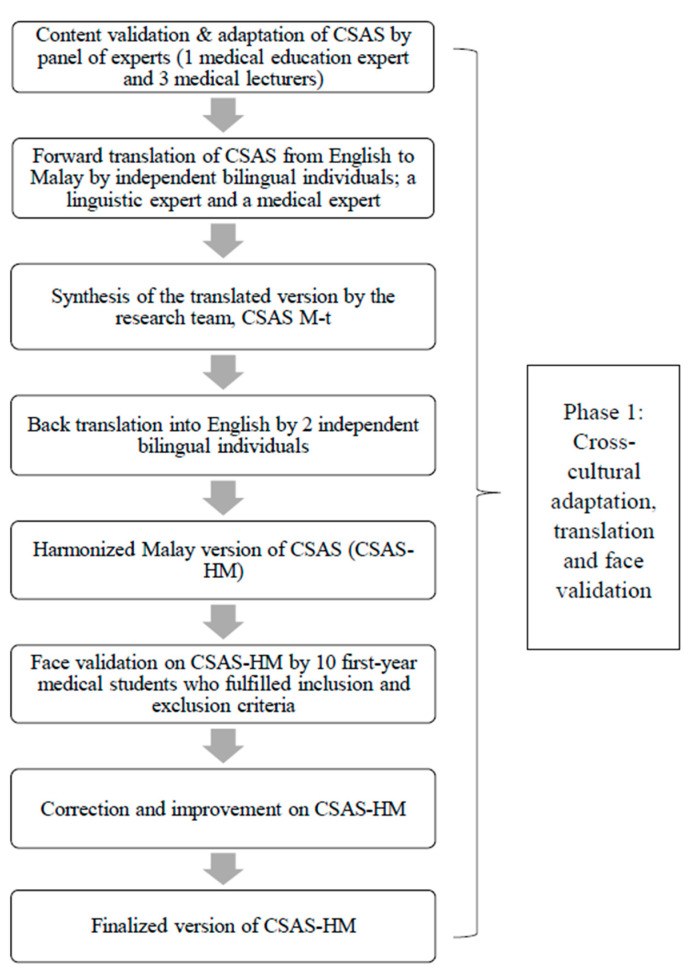

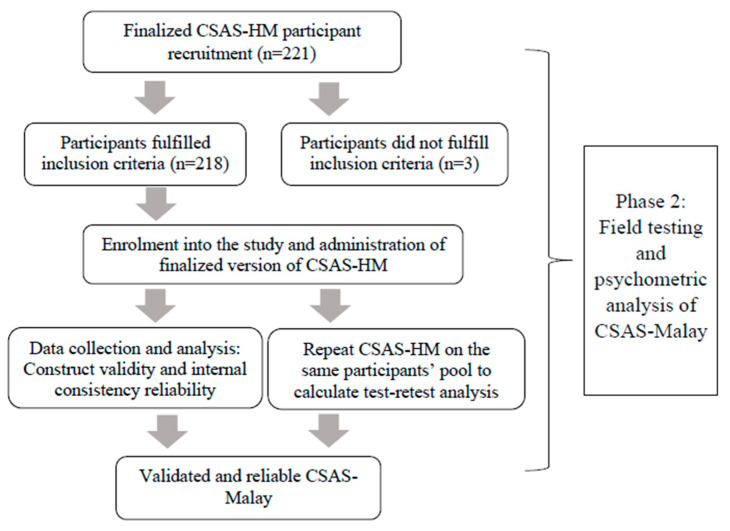
Flowchart of study.

This was a cross-sectional questionnaire validation study conducted in two phases: Phase 1: Cross-cultural adaptation, translation, and face validation; Phase 2: Field testing and psychometric analysis. The flowchart of the study is outlined in Figure 1. First-year medical students from UiTM were chosen in this study because they had no previous formal exposure toward communication skills teaching.

### 2.2. Study Tool

The study tool comprised two parts. Part 1 included demographic details such as age and gender, and education-related items such as type of secondary school attended and the students’ self-rating of their communication skills. Part 2 consisted of the 26-item CSAS (Appendix A), which contains 13 positive statements (PAS) and 13 negative statements (NAS) regarding learning communication skills. The items for the PAS were 4, 5, 7, 9, 10, 12, 14, 16, 18, 21, 22, 23, and 25, and the remainder were for the NAS (1, 2, 3, 6, 8, 11, 13, 15, 17, 19, 20, 24, and 26). All the items had a 5-point Likert scale ranging from 1 (strongly disagree) to 5 (strongly agree). Written permission from the original author of the CSAS English version was obtained to cross-culturally adapt and translate the CSAS into the Malay language.

### 2.3. Conduct of the Study

#### 2.3.1. Phase 1: Cross-Cultural Adaptation, Translation, and Face Validation

The cross-cultural adaptation and translation processes were performed in accordance with the recommendations made in established guidelines for validation studies [25,26]. The number of recommended experts to review an instrument ranges from two to 20 [27]. In this study, the content validation was conducted by a panel of experts, which consisted of an expert in medical education and three medical lecturers. The content experts were nominated based on their expertise and experience in medical education. All of the experts were involved with teaching communication skills to the medical students in their respective universities. They were provided with the CSAS questionnaire and a critical appraisal sheet during the discussion on content validation. They were invited to provide their opinions on the questionnaire based on the objectives of this study. There are a few methods to assess content validity, and this study used empirical and semi-empirical techniques. For the empirical technique, the index of content validity (CVI) was calculated and computed using the item-CVI (I-CVI) and the scale-level-CVI (S-CVI) [28]. The I-CVI expresses the proportion of agreement on the relevancy of each item, which is between zero and one [29]. The content validation experts were asked to rate the relevancy of each item on a scale from 1 to 4. The I-CVI was calculated based on the number of experts rating each item as 3 (quite relevant) or 4 (highly relevant), divided by the total number of experts [30]. Items rated as 3 or 4 were given a score of 1, while items rated as 1 or 2 were given a score of 0. The value of the I-CVI ranged from 0 to 1. According to Lynn et al., if there are fewer than six total experts, items with an I-CVI less than 1 should be eliminated [30]. Similarly, the S-CVI was calculated by using the average CVI (S-CVI/Ave), where the sum of the I-CVI was divided by the total number of items and an acceptable S-CVI value was ≥0.80 [31]. The clarity of the items was discussed using the qualitative content validation method, and the panel was also asked about their suggestions regarding how to improve the items. The discussion points and notes were transcribed by the lead researcher. 

Forward translation of the CSAS was performed by two independent bilingual individuals: One medical expert, who is a family medicine specialist, and one linguistic expert from the UiTM Academy of Language Studies. The CSAS Malay-translated version (CSAS M-t) was produced following a thorough discussion between the research team and both translators via the process of reconciliation. Following this, another two independent bilingual individuals (one medical expert and one linguistic expert from UiTM Academy of Language Studies) back-translated the CSAS M-t from Malay into English without referring to the original CSAS English version. The next step was the harmonization process by the research team. The discussion and comparison of all the translated versions were conducted to finally produce the harmonized CSAS Malay version (CSAS-HM). This CSAS-HM was then used for face validation.

Ten first-year medical students who fulfilled the inclusion and exclusion criteria were recruited for the face validation process. The inclusion criteria were: (i) year 1 medical student intake of 2019, (ii) age > 18 years old, (iii) able to read and understand Malay and (iv) willing to participate and provide informed consent. The exclusion criteria were: (i) year 1 medical student intake of 2018 and (ii) year 2 medical student or above. These students were requested to provide their comments and suggestions regarding the instruction, wording, and overall structure of the questionnaire. These ten students were not re-selected for Phase 2 of this study.

#### 2.3.2. Phase 2: Field Testing and Psychometric Analysis 

The CSAS-HM, which had undergone content validation, translation, and face validation, was then subjected to field testing and psychometric analysis. First-year medical students were recruited from UiTM. The same selection criteria for Phase 1 were applied for recruitment. The sample size for field testing was determined using an item-to-subject ratio ranging from 3:1 to 20:1 [25]. For this study, the ratio of 5:1 was chosen. As the CSAS contains 26 items, the estimated minimum number of participants was 130. However, considering 20% of the non-responder and non-eligibility rates, the target for recruitment was at least 160 participants.

Permission was obtained from the University’s Vice Dean for Academic Affairs, and all the Year 1 students present in a lecture hall on 19 September 2019 were given a brief explanation of the study by the lead researcher. The objectives of the research were explained and instructions on how to complete the questionnaire were provided. Subsequently, the CSAS-HM questionnaires were distributed to all the participants along with an information sheet about the study and a consent form. Participants who fulfilled the selection criteria were requested to complete the questionnaire. The questionnaires and consent forms were then collected and evaluated for completeness. Participants were ensured that their responses would remain confidential, and their data would only be used for this research and would not be shared with anyone outside of the research team. They were not compensated for participating in this study. The students were given a date to repeat the same questionnaire in four weeks’ time. This time interval between the administration of the two questionnaires was considered appropriate as it was long enough to prevent recall but short enough to ensure that no changes in their attitude would have occurred [32].

### 2.4. Statistical Analysis

Data were analyzed using IBM’s Statistical Package for the Social Sciences (SPSS) version 25 (IBM Corp., Armonk, NY, USA). For the descriptive analysis, the data were presented using frequencies and percentages. The construct validity was assessed using principal component analysis (PCA) with direct oblimin rotation. Direct oblimin rotation was chosen instead of promax as the sample size of this study was only 218, which is fewer than the minimum of 300 recommended for the latter method [33]. The adequacy of the sample and data suitability for factor analysis were assessed using the Kaiser–Meyer–Olkin (KMO) test and Bartlett’s test of sphericity, respectively. Data were considered appropriate for analysis when the KMO value was >0.5 (ranging from 0 to 1) and the *p*-value for Bartlett’s test of sphericity was <0.05 [34]. A parallel analysis with a Monte Carlo simulation was also performed. This method identified factors based on randomly generated data that matched the sample size and the number of potential scale items in the original dataset [35]. The internal consistency of the CSAS-Malay was assessed using Cronbach’s α coefficient. A value of >0.7 was considered reliable [34]. The test–retest reliability was assessed using intraclass correlation coefficients (ICC), with values ≥0.7 considered as exhibiting a good reproducibility [36,37].

## 3. Results

### 3.1. Demographic Details of Participants

A total of 221 first-year medical students were approached. Three students did not fulfil the inclusion and exclusion criteria. Therefore, 218 participants were recruited, which amounted to a response rate of 98%. All of the participants completed the questionnaires satisfactorily. The majority of participants were females (72%), Malays (98.2%), and spoke Malay as their first language (93.6%). The demographic details of the participants are presented in Table 1.

### 3.2. Cross-Cultural Adaptation, Translation, and Face Validation

During the discussion in the content validation stage, the I-CVI was calculated to objectively measure the relevance of all the items in the questionnaire. Each item in the CSAS was given an I-CVI value of 1.00 by the panel of experts, except for item 11, which was <1.00. According to Lynn et al., the I-CVI should be more than 1.00 if the number of experts are five or fewer [30]. All four experts agreed to remove item number 11 as the item was perceived to lack relevance and clarity when translated into Malay. The statements of the remaining 25 items had reached a consensus after the expert committee’s discussion, except four items, to which the committee suggested the following changes: (1) Changing the word “point” to “benefit” in item 2, (2) substituting the word “Nobody” to “Medical student” in item 3, (3) changing the word “non-clinical lecturer” to “non-medical lecturer” in item 15, and (4) rephrasing the sentence in item 19, “I don’t need good communication skills to be a doctor” to “Good communication skills are not required to be a doctor”. The remaining 25 items subsequently underwent forward and backward translation in which the bilingual experts and the research team were able to reach a consensus for the translations and produce the CSAS-Malay-harmonized version. During the face validation stage, 10 medical students were recruited. Based on their suggestions, minor corrections were made to improve the quality of the CSAS-HM, such as increasing the font size and including a Likert scale on every page of the questionnaire.

### 3.3. Psychometric Analysis

The measurement of sampling adequacy using the KMO test was 0.795 and Bartlett’s test of sphericity yielded statistical significance with a *p*-value of <0.001. These confirmed the suitability of the data for factor analysis. The initial PCA with direct oblimin rotation revealed nine factors with eigenvalues of more than 1, which accounted for 63.54% of the variance. A scree plot showed an inflexion at Factor 2, suggesting one factor to be retained. A further assessment using parallel analysis with Monte Carlo PCA suggested three factors to be retained. Among the two- to nine-factor solutions examined, a two-factor solution yielded the most interpretable result and was conceptually equivalent to the original scale, explaining 28% of the total variance. This two-factor solution was explored repeatedly by assessing the factor loadings and item performance. Problematic items were eliminated in a stepwise process. Two items were removed one by one as they had a poor factor loading (<0.300). The removed items were Q15 and Q1. The final factor solution obtained consisted of 23 items loading on two factors and was conceptually equivalent to the original scale, explaining 30.8% of the total variance. At this point, item 13 was cross-loaded on both factors. This item conceptually fit into Factor 2 and was, therefore, retained in this factor. Table 2 shows the final rotated pattern matrix based on a two-factor solution, after the removal of items Q15 and Q1. 

Based on the pattern matrix, items that loaded onto Factor 1 (4, 5, 9, 10, 12, 14, 16, 18, 21, 23, and 25) were positive statements, while items that loaded onto Factor 2 (2, 3, 6, 7, 8, 13, 17, 19, 20, 22, 24, and 26) were negative statements, except for items 7 and 22. Thus, Factor 1 was labeled as the Positive Attitude Scale (PAS) and Factor 2 was labeled as the Negative Attitude Scale (NAS).

In summary, one item (Q11) was removed during content validation while two items (Q15 and Q1) were removed following factor analysis, leaving a total of 23 items framed into two subscales (PAS and NAS) for the CSAS-Malay. The final PAS consisted of 11 items (4, 5, 9, 10, 12, 14, 16, 18, 21, 23, and 25) and the final NAS consisted of 12 items (2, 3, 6, 7, 8, 13, 17, 19, 20, 22, 24, and 26).

### 3.4. Reliability

A total of 23 items were included for internal consistency testing with 11 items for the PAS and 12 items for the NAS. Item 7 loaded negatively onto the NAS; therefore, it was reverse-coded in the calculation of the internal consistency of the NAS. Cronbach’s α coefficients for both the PAS and NAS in the final CSAS-Malay were 0.815 and 0.614, respectively. Table 3 shows Cronbach’s α values for both subscales, as well as the intraclass correlation coefficients (ICCs) for each item. A value ≥0.7 for the correlation coefficient is considered good [36,37]. ICC values can be further classified into less than 0.5, 0.5 to 0.75, 0.75 to 0.9, and more than 0.9, indicating poor, moderate, good, and excellent reliabilities, respectively [38]. Based on this definition, the ICC values for all of the CSAS-Malay items showed good reliabilities, except for items 12, 16, and 20, which showed moderate reliabilities, and item 19, which showed an excellent reliability.

## 4. Discussion

To the best of our knowledge, this is the first study to cross-culturally adapt, translate and validate a tool in the Malay language to assess the attitude of Malaysian medical students toward learning communications skills. The processes of cross-cultural translation and adaptation of the original version of the CSAS were meticulously followed to produce the CSAS-Malay (Appendix B). This study provided preliminary information about the psychometric properties of the CSAS-Malay. The final 23-item CSAS-Malay was found to have a good construct validity, internal consistency, and test–retest reproducibility. 

During content validation, item 11 (“Communication skills teaching states the obvious and then complicates it”) from the original CSAS was removed by all four members of the panel of experts as they perceived that the item may give rise to an ambiguous understanding by the medical students. This decision was also consistent with the I-CVI of this item, which was less than 1.00. This item, however, has been retained in other CSAS translation and validation studies [8,9,11,19,20,21,22,23].

The CSAS-Malay consisted of two subscales, which is similar to the original CSAS and other translated versions [8,9,13,22]. Previous translation and validation studies of the CSAS showed a variety of subscales and item compositions for each subscale, as illustrated in Table 4. Items 7 and 22, which were positive statements based on the original study, were found to be grouped under the NAS in the CSAS-Malay after undergoing factor analysis. These differences may be contributed by different understandings and interpretations of each question after being translated into various languages. Zhang et al. suggested that the attitude of medical students toward each item may be inconsistent as this could be influenced by different cultural backgrounds and variations in the processes used to develop the communication skills curricula [23].

The findings from this study support the hypothesis that the psychometric properties of the scale would improve if adapted and translated into the Malay language and administered among students who speak Malay as their first language. This is evident from the improved internal consistency of the NAS in the CSAS-Malay (α = 0.614), as compared to the previous study by Baharudin et al. (α = 0.565) [24]. However, compared to the other studies, the internal consistency of the NAS in the CSAS-Malay is lower but still acceptable (Table 4) [8,9,11,13,19,20,21,22,23]. It is proposed that any change, including the translation of words, in a research tool will have some influence on the measurement and interviewee [19]. Compared to the original CSAS and other translated versions (Table 4), the internal consistency of the PAS in the CSAS-Malay is lower (α = 0.815) but acceptable [8,11,13,20,22]. 

### 4.1. Strengths and Limitations

The strength of this study was the universal sampling method as it minimized selection bias. However, the study sample was limited to a university with a majority of Malay-speaking students. This study population might not be representative of all the medical students in Malaysia. Hence, the use of the CSAS-Malay among other medical schools in Malaysia may require further adaptation and validation as their population may vary. 

During the content validation stage, one item was removed as the expert panel agreed that the statement may be perceived as ambiguous and confusing to the medical students. However, the expert panel in this study consisted of medical lecturers. Medical students could be included as the lay experts during the content validation stage to obtain their suggestions and opinions regarding the items in the original CSAS questionnaire. The presence of lay experts may ensure that the population for whom the instrument is being developed is represented [39]. The use of a scoring system for the clarity for each item based on a four-point ordinal scale (1 (not clear), 2 (item needs some revision), 3 (clear but needs some revision), 4 (very clear)) should also be included during the content validation stage in future studies to improve the quality of the translation process [29].

### 4.2. Implications on Curriculum Improvement & Future Research

The results of the psychometric properties of the CSAS-Malay from this study suggest that it can be utilized in practice to measure the attitudes of students toward learning communication skills in predominantly Malay-speaking medical students such as in UiTM. Objective measurements of the attitudes of students toward learning this subject matter is vital to guide the Faculty in improving the communication skills program. Future research may include the sampling of Year 2 to 5 students, and students from other universities, to improve its generalizability. Confirmatory factor analysis should also be conducted to verify the two-factor structure of the CSAS-Malay. Future research may also include the identification of the factors associated with poor attitude so that appropriate measures can be implemented to address this. The CSAS-Malay can also be used to measure the effectiveness of communication skills teaching in improving the attitudes of students toward learning these skills. This can be achieved by using the measurement of the attitudes of medical students before and after undergoing a communication skills program. These findings would guide curriculum planners in the development and improvement of this program.

## 5. Conclusions

This validation study provided preliminary information about the psychometric properties of the CSAS-Malay. The final 23-item CSAS-Malay had a good construct validity, an acceptable internal consistency, and at least a moderate test–retest reproducibility. It can be used to assess the attitudes of UiTM medical students toward learning communication skills. To improve the generalizability of the CSAS-Malay, future research should include medical students from other universities with diverse backgrounds. To confirm the construct of the CSAS-Malay, a further evaluation with confirmatory factor analysis (CFA) should be performed.

## Figures and Tables

**Table 1:** Demographic details of participants.

Characteristics of Participants	Total (*n* = 218) *N* (%)	Median (Interquartile Range)
**Age** (years)		19(0.00)
19	205(94)	
20	3(1.4)	
21	8(3.7)	
23	1(0.5)	
25	1(0.5)	
**Gender**		
Male	61(28)	
Female	157(72)	
**Ethnicity**		
Malay	214(98.2)	
Non-Malay	4(1.8)	
**Family monthly household income ^1^**		
>MYR13148	50(22.9)	
MYR3001-13147	106(48.7)	
<MYR3001	54(24.8)	
Not known	8(3.7)	
**First language**		
Malay	204(93.6)	
English	13(6.0)	
Others	1(0.4)	
**Self-rating on communication skills**		
Very good	11(5.0)	
Good	119(54.6)	
Satisfactory	69(31.7)	
Weak	18(8.3)	
Very weak	1(0.5)	
**Do students think their communication skills need improving**		
Yes	217(99.5)	
No	1(0.5)	

^1^ MYR: Malaysian Ringgit.

**Table 2:** Final rotated pattern matrix with 23 items (Q15 and Q1 removed).

Items	Questions	Positive (P)/ Negative (N) Scale	Factor	
			1	2
Q14	Learning communication skills has helped or will help me respect my colleagues	P	**0.740**	−0.077
Q10	Learning communication skills has improved my ability to communicate with patients	P	**0.645**	−0.147
Q9	Learning communication skills has helped or will help facilitate my team-working skills	P	**0.673**	−0.366
Q21	I think it’s really useful learning communication skills on the medical degree	P	**0.662**	−0.324
Q23	Learning communication skills is applicable to learning medicine	P	**0.616**	−0.065
Q16	Learning communication skills has helped or will help me recognise patients’ rights regarding confidentiality and informed consent	P	**0.597**	−0.140
Q25	Learning communication skills is important because my ability to communicate is a lifelong skill	P	**0.554**	0.143
Q5	Learning communication skills has helped or will help me respect patients	P	**0.531**	−0.308
Q18	When applying for medicine, I thought it was a really good idea to learn communication skills	P	**0.517**	−0.404
Q4	Developing my communication skills is just as important as developing my knowledge of medicine	P	**0.485**	−0.314
Q12	Learning communication skills is fun	P	**0.474**	−0.274
Q26	Communication skills learning should be left to psychology students, not medical students	N	−0.263	**0.371**
Q24	I find it difficult to take communication skills learning seriously	N	−0.207	**0.553**
Q22	My ability to pass exams will get me through medical school rather than my ability to communicate	P	−0.149	**0.497**
Q8	I can’t be bothered to turn up to sessions on communication skills	N	0.116	**0.518**
Q2	I can’t see the point in learning communication skills	N	−0.284	**0.488**
Q3	Nobody is going to fail their medical degree for having poor communication skills	N	−0.083	**0.436**
Q20	I find it hard to admit to having some problems with my communication skills	N	0.032	**0.420**
Q19	I don’t need good communication skills to be a doctor	N	−0.251	**0.490**
Q6	I haven’t got time to learn communication skills	N	−0.117	**0.370**
Q13	Learning communication skills is too easy	N	**0.382**	**0.335**
Q7	Learning communication skills is interesting	P	0.294	**−0.324**
Q17	Communication skills teaching would have a better image if it sounded more like a science subject	N	0.159	**0.304**

Bold: Items are emboldened to indicate the significant factor in which they belong.

**Table 3:** Cronbach’s α coefficients for the Positive Attitude Scale (PAS) and Negative Attitude Scale (NAS).

Subscale	Cronbach’s Alpha	Items	Corrected Item-Total Correlation	Cronbach’s Alpha If Item Deleted	Intraclass Correlation Coefficient (ICC (95%CI))
PAS	0.815	Q4	0.443	0.805	0.853 (0.699–0.929)
		Q5	0.442	0.804	0.783 (0.555–0.895)
		Q9	0.601	0.792	0.807 (0.599–0.907)
		Q10	0.526	0.798	0.882 (0.747–0.944)
		Q12	0.393	0.815	0.737 (0.449–0.874)
		Q14	0.576	0.791	0.915 (0.822–0.959)
		Q16	0.457	0.803	0.725 (0.425–0.868)
		Q18	0.484	0.801	0.856 (0.703–0.931)
		Q21	0.566	0.793	0.754 (0.490–0.881)
		Q23	0.486	0.800	0.736 (0.452–0.873)
		Q25	0.437	0.805	0.783 (0.556–0.889)
NAS	0.614	Q2	0.442	0.598	0.878 (0.749–0.941)
		Q3	0.302	0.604	0.846 (0.680–0.926)
		Q6	0.267	0.611	0.811 (0.607–0.909)
		Q8	0.353	0.592	0.820 (0.630–0.913)
		Q13	0.031	0.636	0.775 (0.551–0.891)
		Q17	0.114	0.630	0.809 (0.605–0.907)
		Q19	0.353	0.600	0.950 (0.898–0.976)
		Q20	0.241	0.619	0.726 (0.431–0.868)
		Q22	0.346	0.594	0.777 (0.492–0.897)
		Q24	0.405	0.579	0.752 (0.494–0.880)
		Q26	0.356	0.599	0.764 (0.510–0.887)
		Q7(rev)	0.225	0.618	0.852 (0.696–0.928)

**Table 4:** Subscales and internal consistency values in translated versions of the Communication Skills Attitude Scale (CSAS).

Author [Reference]	Country	Language	Subscale	Cronbach’s Alpha	Items
Rees et al. [13]	United Kingdom	English	Positive attitude scale	0.873	4,5,7,9,10,12,14,16,18,21,22,23,25
			Negative attitude scale	0.805	1,2,3,6,8,11,13,15,17,19,20,24,26
Mohamad Isa et al.	Malaysia	Malay	Positive attitude scale	0.815	4,5,9,10,14,16,18,20,21,23,25
(this study)			Negative attitude scale	0.614	2,3,6,7,8,13,17,18,19,24,22,26
Zhang et al. [23]	China	Chinese	Importance of communication skills	0.771	1,4,5,9,10,14,16,21,25
			Negative beliefs	0.601	2,6,8,11,13,15,19,24,26
			Motivation	0.637	12,17,23
			Assessment	0.704	3,20,22
Busch et al. [8]	Germany	German	Positive attitude scale	0.864	4,5,9,10,14,16,23
			Negative attitude scale	0.838	2,6,7,11,12,15,17,19,21,24,25,26
Koponen et al. [21]	Finland	Finnish	Positive attitude scale	0.882–0.895	4,5,7,9,10,12,14,16,18,21,22,23,25
			Negative attitude scale	0.794–0.828	1,2,3,6,8,11,13,15,17,19,20,24,26
Molinuevo et al. [22]	Spain	Catalan	Positive attitude scale	0.83	1,4,5,7,9,10,12,14,16,18,21,23,25
			Negative attitude scale	0.64	2,3,6,8,11,15,17,20,22,24
Ihmeideh et al. [20]	Jordan	Arab	Positive attitude scale	0.87	4,5,7,9,10,12,14,16,18,21,22,23,25
			Negative attitude scale	0.81	1,2,3,6,8,11,13,15,17,19,20,24,26
Ahn et al. [19]	Korea	Korean	Facilitating interpersonal skill	0.752	4,5,9,10,14,16
			Importance within medical context	0.744	2,19,21,26
			Motivation	0.68	8,11,23,24
			Assessment	0.446	3,22
			Overconfidence	0.496	13,20
Harlak et al. [9]	Turkey	Turkish	Positive attitude scale	0.92	1,4,5,7,8,9,10,12,13,14,16,18,21,23,25
			Negative attitude scale	0.71	2,3,6,11,15,17,19,20,22,24,26
Anvik et al. [11]	Norway	Norwegian	Learning	0.861	2,6,7,8,10,11,12,13,18,21,24,25,26
			Importance	0.532	1,3,4,19,22
			Respecting	0.775	5,9,14,16

## Data Availability

Data are kept at the Department of Primary Care Medicine, Universiti Teknologi MARA, in Selangor, Malaysia. Data will be shared upon request and they are subjected to the data protection regulations.

## Appendix A. CSAS Original

1. In order to be a good doctor I must have good communication skills
2. I can’t see the point in learning communication skills
3. Nobody is going to fail their medical degree for having poor communication skills
4. Developing my communication skills is just as important as developing my knowledge of medicine
5. Learning communication skills has helped or will help me respect patients
6. I haven’t got time to learn communication skills
7. Learning communication skills is interesting
8. I can’t be bothered to turn up to sessions on communication skills
9. Learning communication skills has helped or will help facilitate my team-working skills
10. Learning communication skills has improved my ability to communicate with patients
11. Communication skills teaching states the obvious and then complicates it
12. Learning communication skills is fun
13. Learning communication skills is too easy
14. Learning communication skills has helped or will help me respect my colleagues
15. I find it difficult to trust information about communication skills given to me by non-clinical lecturers
16. Learning communication skills has helped or will help me recognize patients’ rights regarding confidentiality and informed consent
17. Communication skills teaching would have a better image if it sounded more like a science subject
18. When applying for medicine, I thought it was a really good idea to learn communication skills
19. I don’t need good communication skills to be a doctor
20. I find it hard to admit to having some problems with my communication skills
21. I think it’s really useful learning communication skills on the medical degree
22. My ability to pass exams will get me through medical school rather than my ability to communicate
23. Learning communication skills is applicable to learning medicine
24. I find it difficult to take communication skills learning seriously
25. Learning communication skills is important because my ability to communicate is a lifelong skill
26. Communication skills learning should be left to psychology students, not medical students

## Appendix B. CSAS-Malay

1. Saya tidak nampak kepentingan mempelajari kemahiran komunikasi
2. Pelajar perubatan tidak akan gagal dalam memperoleh ijazah perubatan kerana mempunyai kemahiran komunikasi yang lemah
3. Menguasai kemahiran komunikasi adalah sama pentingnya dengan menguasai ilmu perubatan
4. Mempelajari kemahiran berkomunikasi telah membantu atau akan membantu saya untuk menghormati pesakit
5. Saya tiada masa untuk mempelajari kemahiran komunikasi
6. Mempelajari kemahiran komunikasi adalah menarik
7. Saya tidak peduli untuk menghadiri kelas kemahiran komunikasi
8. Mempelajari kemahiran komunikasi telah membantu atau akan menambahkan kemahiran saya bekerja secara berpasukan
9. Mempelajari kemahiran komunikasi telah menambah baik kebolehan saya berkomunikasi dengan pesakit
10. Mempelajari kemahiran komunikasi adalah menyeronokkan
11. Mempelajari kemahiran komunikasi terlalu mudah
12. Mempelajari kemahiran komunikasi telah atau akan membantu saya menghormati rakan sekerja
13. Saya berasa sukar untuk menerima ilmu kemahiran komunikasi yang diajar oleh pensyarah bukan dari bidang perubatan
14. Pengajaran kemahiran komunikasi akan lebih menarik jika ia disampaikan seperti subjek sains
15. Semasa memohon kursus perubatan, saya fikir mempelajari kemahiran komunikasi adalah idea yang sangat baik
16. Kemahiran komunikasi yang baik tidak diperlukan untuk menjadi seorang doctor
17. Saya berasa sukar untuk mengakui terdapat sedikit masalah dengan kemahiran komunikasi saya
18. Saya berpandangan mempelajari kemahiran komunikasi sangat berguna semasa kursus perubatan
19. Keupayaan saya untuk lulus peperiksaan akan memastikan saya lulus jurusan perubatan dan bukannya kemampuan saya berkomunikasi
20. Mempelajari kemahiran komunikasi boleh diaplikasikan dalam mempelajari ilmu perubatan
21. Saya berasa sukar untuk menganggap pembelajaran kemahiran komunikasi sebagai sesuatu yang serius
22. Mempelajari kemahiran komunikasi sangat penting kerana kebolehan saya berkomunikasi merupakan kemahiran sepanjang hayat
23. Kemahiran komunikasi sepatutnya hanya dipelajari oleh pelajar psikologi, bukannya pelajar perubatan
